# Induction of Indoleamine 2, 3-Dioxygenase in Human Dendritic Cells by a Cholera Toxin B Subunit—Proinsulin Vaccine

**DOI:** 10.1371/journal.pone.0118562

**Published:** 2015-02-25

**Authors:** Jacques C. Mbongue, Dequina A. Nicholas, Kangling Zhang, Nan-Sun Kim, Brittany N. Hamilton, Marco Larios, Guangyu Zhang, Kazuo Umezawa, Anthony F. Firek, William H. R. Langridge

**Affiliations:** 1 Center for Health Disparities and Molecular Medicine, Department of Basic Sciences, Loma Linda University School of Medicine, Loma Linda, CA, United States of America; 2 Loma Linda University School of Medicine, Department of Basic Sciences, Division of Physiology, Loma Linda, CA, United States of America; 3 Mass Spectrometer Core Facility, Department of Biochemistry, Loma Linda University School of Medicine, Loma Linda University School of Medicine, Department of Basic Sciences, Loma Linda, CA, United States of America; 4 Department of Pharmacology and Toxicology, School of Medicine, University of Texas Medical Branch, Galveston, TX, United States of America; 5 Loma Linda University School of Medicine, Department of Basic Sciences, Division of Microbiology and Molecular Genetics, Loma Linda, CA, United States of America; 6 Aichi Medical University, School of Medicine, Department of Molecular Target Medicine Screening, Nagakute, Aichi, Japan; 7 Endocrinology Section, JL Pettis Memorial VA Medical Center, Loma Linda, CA, United States of America; 8 Department of Molecular Biology, Chonbuk National University, Jeon-Ju, Republic of Korea; Université Paris Descartes, FRANCE

## Abstract

Dendritic cells (DC) interact with naïve T cells to regulate the delicate balance between immunity and tolerance required to maintain immunological homeostasis. In this study, immature human dendritic cells (iDC) were inoculated with a chimeric fusion protein vaccine containing the pancreatic β-cell auto-antigen proinsulin linked to a mucosal adjuvant the cholera toxin B subunit (CTB-INS). Proteomic analysis of vaccine inoculated DCs revealed strong up-regulation of the tryptophan catabolic enzyme indoleamine 2, 3-dioxygenase (IDO1). Increased biosynthesis of the immunosuppressive enzyme was detected in DCs inoculated with the CTB-INS fusion protein but not in DCs inoculated with proinsulin, CTB, or an unlinked combination of the two proteins. Immunoblot and PCR analyses of vaccine treated DCs detected *IDO1*mRNA by 3 hours and IDO1 protein synthesis by 6 hours after vaccine inoculation. Determination of IDO1 activity in vaccinated DCs by measurement of tryptophan degradation products (kynurenines) showed increased tryptophan cleavage into N-formyl kynurenine. Vaccination did not interfere with monocytes differentiation into DC, suggesting the vaccine can function safely in the human immune system. Treatment of vaccinated DCs with pharmacological NF-κB inhibitors ACHP or DHMEQ significantly inhibited IDO1 biosynthesis, suggesting a role for NF-κB signaling in vaccine up-regulation of dendritic cell IDO1. Heat map analysis of the proteomic data revealed an overall down-regulation of vaccinated DC functions, suggesting vaccine suppression of DC maturation. Together, our experimental data indicate that CTB-INS vaccine induction of IDO1 biosynthesis in human DCs may result in the inhibition of DC maturation generating a durable state of immunological tolerance. Understanding how CTB-INS modulates IDO1 activity in human DCs will facilitate vaccine efficacy and safety, moving this immunosuppressive strategy closer to clinical applications for prevention of type 1 diabetes autoimmunity.

## Introduction

The continued failure to find a cure for type 1 diabetes mellitus (T1D) is a prime example of the urgent need for therapeutics that can safely deliver antigen specific therapy for protection against tissue specific autoimmunity. Type 1 diabetes is a well-known prototypic tissue specific autoimmune disease that results from auto-reactive lymphocyte destruction of the pancreatic islet insulin-producing β-cells [1,2]. The progressive loss of islet β-cell function leads to insulin deficiency and high blood glucose levels (hyperglycemia). Increased cellular oxidative stress and chronic inflammation generated by hyperglycemia can result in neural and circulatory complications leading to amputation, loss of kidney function, blindness, heart attack, and stroke resulting in early mortality [3,4].

Dendritic cells, considered the most prominent subset of professional antigen presenting cells (APC), are implicated in islet β-cell destruction [5–7]. Through presentation of pancreatic β-cell antigens to naïve auto-reactive T helper cells, DCs can guide their differentiation into effector Th1 and Th17 lymphocytes capable of killing pancreatic β-cells [2,8–10]. Auto-reactive CD4^+^ Th1 lymphocytes were shown to infiltrate pancreatic islets in non-obese diabetic (NOD) mice and secrete the pro-inflammatory cytokines IFN-γ and IL-2 [11–13]. Dendritic cell regulation of T cell morphogenesis is required for maintenance of immunological homeostasis. However, in the presence of auto-reactive T cells, DC presentation of β-cell auto-antigens may stimulate activation of these T cells and disrupt the delicate balance between immune stimulation and immune suppression. The consequent imbalance can result in development of chronic inflammation that may progress to T1D.

Immunological suppression strategies including combinatorial vaccines that link immune-stimulatory molecules (adjuvants) to antigens, have been developed to enhance vaccine efficacy [14–16]. Prominent among adjuvants is the cholera toxin B-subunit (CTB) [17]. Linkage of CTB to insulin (CTB-INS) results in a fusion protein that has been shown to have a protective effect against the onset of T1D [16]. Initial oral immunization experiments showed that feeding small amounts (2–20μg) of CTB-INS could effectively suppress β-cell destruction and clinical diabetes in adult NOD mice [14,18]. Initial recognition of the mechanism underlying vaccine mediated immune suppression was based on CTB-INS induction of CD4^+^ regulatory T cells (Tregs) in NOD mice [19]. Prediabetic non-obese diabetic (NOD) mice inoculated with CTB-INS fusion protein showed a decrease in pancreatic islet inflammation (insulitis) characterized by a reduction in islet infiltration with auto-reactive lymphocytes, increased proliferation of CD4^+^ regulatory T-cells, and suppression of T1D onset [16,19–21]. In addition, increased expression of anti-inflammatory cytokines, such as interleukin-4 (IL-4) and interleukin-10 (IL-10), were detected in pancreatic tissue isolated from treated animals [22]. This work provided a platform for more recent experiments that showed CTB-INS inhibition of dendritic cell maturation can be identified by vaccine suppression of CD86 and CD80 co-stimulatory factor biosynthesis [23,24]. Demonstrating the broad range of applications of this vaccine strategy for suppression of autoimmunity, conjugation of CTB with islet auto-antigens such as insulin and glutamic acid decarboxylase (GAD) was shown to induce immunological tolerance through the suppression of DC maturation [18,24]. Further, fusion of an uveitogenic HSP60 derived-peptide (aa 336–351) with CTB resulted in significant protection against mucosally induced uveitis and Behcet’s disease [25,26]. Of further importance, fusion of CTB with myelin oligodendrocyte glycoprotein (MOG) provided protection against the development of multiple sclerosis [27].

To date however, the molecular mechanism responsible for vaccine stimulation of DC mediated immunosuppressive responses remains unknown. Thus, before effective chimeric vaccine immune suppression strategies can be applied in clinical studies, mechanisms underlying vaccine suppression of DC maturation must be identified and are the subject of experiments described in this study.

## Materials and Methods

### Construction of bacterial expression vectors containing the cholera toxin B subunit—proinsulin gene fusion

A DNA sequence encoding 258bp of the human proinsulin gene (INS M12913.1) was linked to the carboxyl-terminus of a DNA fragment (309bp) encoding the cholera toxin B subunit gene (CTB U25679.1) to generate the fusion gene CTB-INS. Four GpGp sequences were inserted between the two genes to promote molecular flexibility [28]. The CTB-INS fusion gene was cloned into *E*. *coli* expression vector PBR-322 and the plasmid amplified in *E*. *coli* HB101 [29]. To achieve high levels of transgene expression, the CTB-INS gene fusion was subcloned into *E*. *coli* expression vector pRSET-A (Invitrogen, Carlsbad, CA) under control of the bacteriophage T7 promoter [23]. The resultant bacterial expression vector (pRSET-CTB-INS) contains an oligonucleotide encoding 6 contiguous histidines located immediately upstream of CTB-INS to permit nickel affinity column isolation of the recombinant fusion protein. Expression vector pRSET-CTB-INS was transformed into the *E*. *coli* producer strain BL21 (DE3) pLysS (Invitrogen, Carlsbad, CA) for production and isolation of milligram amounts of the CTB-INS protein for further experiments [29].

### Synthesis and isolation of CTB-INS fusion protein

The *E*. *coli* strain BL21 transformed with pRSET-CTB-INS [23] was grown overnight at 37°C in a 2.0 ml Luria Broth (LB) shake culture containing 100 μg/ml ampicillin for selection of transformed cells. The lag phase cells were transferred (1.0 ml) into 250 ml Luria Broth (LB) containing ampicillin (100 μg/ml) and cultured at 37°C with gentle shaking. While still in log phase of growth, synthesis of the CTB-INS protein was stimulated by addition of 2 mM fresh isopropyl β-D-1-thiogalacto-pyranoside (IPTG) (Sigma Chemical Co. St. Louis, MO) to the bacterial culture. After a 6-hour continued growth at 37°C, the bacterial culture was transferred into 40 ml polystyrene Oakridge tubes and harvested by centrifugation in a Sorvall SA-600 rotor at 5,000 rpm for 10 minutes at 4°C with the brake off in a Sorvall RC5B centrifuge. The soft cell pellets were resuspended in 1.0 ml of lysis buffer (100 mM HEPES, protease inhibitor-Sigma Complete protease inhibitor cocktail, 5μl/ml DNAse I), (Promega Inc.). The bacteria were disrupted by sonication with 3 × 10 seconds bursts at a setting of 10 W with a Sonic 60 Dismembrator (Fisher Sci. Sunnyvale, CA). The CTB-INS protein was isolated and purified from the bacterial homogenate by nickel affinity chromatography using a Maxwell Model 16 robotic protein purification system (Promega Inc.) according to the protein isolation protocol provided by the manufacturer. The recombinant protein product was isolated on Magnet-His Nickel-Iron alloy particles with an affinity for the 6-HIS tag linked to the N terminus of the recombinant CTB-INS fusion protein. To isolate the protein from the nickel-iron alloy particles, the Ni^+^ particles were transferred into a clean tube and centrifuged at 2000 rpm on an Eppendorf centrifuge equipped with a 5417C rotor at room temperature. The beads were then washed three times with 1.0 mL HEPES (100 mM). The protein was separated from the beads by re-suspension in 1.0 mL Z- buffer (8M Urea, 100 mM NaCl, and 20 mM HEPES, pH 8.0). The mixture was centrifuged for 5 minutes (10,000 rpm, 4°C). Imidazole and urea were removed from the protein by dialysis of the preparation against 2 × 1.0 Liter 10 mM HEPES buffer (pH 7.5) for a total of 4 hours at 4°C. The purity of the CTB-INS protein (23.4 kDa) was determined based on its electrophoretic mobility in a 12% polyacrylamide gel in comparison with protein molecular weight standards (BioRad, CA) [23].

### Ethics

Experiments on monocyte-derived DCs were performed *ex-vivo*, with aphaeresis blood provided by the Life Stream Blood Bank (San Bernardino, CA) with Loma Linda University IRB and blood donor consent. Blood donor information was anonymized and de-identified prior to the study.

### Isolation and culture of monocyte—derived dendritic cells from Human peripheral blood

Monocyte-derived dendritic cells (MoDCs) were prepared from freshly collected human peripheral blood cells isolated from aphaeresis filter cones obtained from the LifeStream blood bank (San Bernardino, CA). The blood was incubated with a red blood cell lysis buffer (3.0 mL Lysis Buffer/ mL of blood) (Boston Bioproducts), containing 8.3g/L NH_4_Cl, 1g/L KHCO_3_, and 1.8 mL 5% EDTA and centrifuged for 5 minutes at 1,500 rpm at 4°C in a Beckman Coulter Allegra X-15R centrifuge, equipped with a SX4750 rotor. The CD14^+^ monocytes were obtained from the total lymphocyte fraction by incubation with anti-CD14 antibodies bound to magnetic beads for 15 minutes at 4°C (Miltenyi Biotech, Auburn, CA). The monocytes were separated from other immune cells by binding to a magnetic MACS column followed by elution of all other leucocytes (Miltenyi Biotech, Auburn, CA). The monocytes were eluted from the column and cultured ranging from 2–9 × 10^6^ cells/well in 6-well non-pyrogenic polystyrene culture plates in RPMI 1640 culture medium (Mediatech Inc. Manassas, VA, USA), at 37°C in a humidified atmosphere of 5% CO_2_ (Preprotech, Rocky Hill, NJ). The medium was supplemented with 10% FBS, 1 mM glutamine, 100 U/ml penicillin, 100 μg/ml streptomycin, 50 ng/ml human recombinant GMCSF, and 10 ng/ml human recombinant IL-4 (ProSpec-Tany TechnoGene, Rehovot Science Park, Israel). The monocyte cell culture was fed at 2-day intervals by gentle replacement of 50% of the medium with fresh culture medium. The cells were cultured for 6 days to allow differentiation into DCs prior to vaccine treatment. The cells were observed by phase contrast microscopy to assess dendrite formation, a marker for DC differentiation.

### Vaccinated dendritic cell sample preparation and mass spectrometric analysis

After treatment with 10 μg/ml of CTB-INS, the dendritic cell pellet was lysed on ice for 2 hours in radio-immunoprecipitation assay (RIPA) buffer (Santa Cruz Biotechnology, CA) containing 1% Nonidet P40, PMSF (0.2 mM), and protease inhibitor cocktail (Roche). The protein mixture was centrifuged at 10,000 rpm at 4°C for 15 minutes in a Beckman GS-15R centrifuge equipped with a F2402 rotor and the supernatant transferred into a clean tube. The protein concentration in the supernatant was determined by BCA Protein Assay (Thermo Scientific Pierce, Rockford, IL USA). Approximately 60 μg of each protein sample was resuspended in 25mM triethylammonium bicarbonate buffer, pH 7.8. The protein was reduced by addition of 10 mM DTT and incubated at 50°C for 30 minutes, followed by carboxymethylation achieved by addition of 25 mM iodoacetamide and incubation of the mixture in the dark for 1 hour. The proteins were precipitated by addition of 4 volumes of precooled (-20°C) acetone and stored at -20°C overnight. The protein was pelleted at 14,000 rpm in the Beckman GS-15R centrifuge for 10 minutes at 4°C and the supernatant discarded. The protein pellet was dissolved in 25 mM triethylammonium bicarbonate buffer (100 μM GTP, 100 μM GDP in 8 mM PIPES pH 6.8) and partially digested by trypsin (Sigma-Aldrich, St. Louis, MO) at a protein / trypsin enzyme ratio of 25:1 (by mass) for 10 hours at 37°C.

A Tandem Mass Tagging isobaric (TMT) Kit (Thermo-Fisher Scientific) was used to label the peptides following the manufacturer’s recommended conditions. Each TMT-labeled protein pool was acidified with 0.1% Formic Acid (FA) and fractionated by strong cation-exchange (SCX) chromatography on a Toptip column (Poly LC, MD). For fractionation, the column matrix was equilibrated with 0.1% FA in 20% acetonitrile (ACN) to facilitate peptide binding. After collection of the flow-through volume, 1.0 mL of each sub-fraction was eluted sequentially with 20% ACN, 0.05 M KCl, 0.2 M KCl, 0.5 M KCL, and 5% ammonium hydroxide in 20% ACN. Next, the fractions were dried under vacuum to remove the ACN, reconstituted in 1% formic acid, and desalted using a Toptip column with C18/hypercarb mixed materials (Poly LC, MD). The eluted peptides were once again vacuum-dried, reconstituted in 30 μl of 0.1% FA, and subjected to LC-MS/MS analysis. The peptides were separated by online reversed phase liquid chromatography (RPLC) using an Easy-nLC equipped with an auto-sampler (Thermo Scientific). A 10 cm, 75 μm id, 3-μm particle size, C18-A2 analytical column (Thermo Scientific) was used for the RPLC separations. Quantitation of the SCX fractionated TMT-6 labeled peptides was carried out on the Thermo LTQ-Orbitrap Velos Pro mass spectrometer. Approximately 2 μg of peptide sample was injected in the analytical column. A pre-column (Thermo, 0.1 × 2 cm, 5 μm C18-A1) was brought in line with the analytical column and a 250-min gradient (solvent A, 0.1% FA in water; solvent B, 0.1% FA in ACN) from 5–30% solvent B was used for separating the peptides. The Orbitrap mass analyzer was set to acquire data at R = 60,000 resolution for the parent full-scan mass spectrum, followed by data-dependent high collision-energy dissociation (HCD) MS/MS spectra for the top 12 most abundant ions acquired at R = 7500 resolution.

### Dendritic cell proteome data analysis

Vaccinated DC proteins were identified and quantified by analysis with the Proteome Discoverer 1.4 platform (Thermo) and the Mascot search engine (Mascot Darmon 2.2.2; Matrix Science, London, UK) employing the International Protein Index (IPI) *Homo sapiens* database (version 3.73, June 2010, containing 89739 entries). Mascot searching parameters were used as follows: Carbamidomethylation of cysteine and TMT-6 modification of the peptide N-terminus and lysine were set as fixed modifications and oxidation of methionine and deamination of asparagine and glutamine were set as variable modifications. Trypsin was the protease selected and preparations containing up to two missed cleavages were used. Mass tolerance for the precursor ions was 10 ppm and for the MS/MS 0.2 Da. The peptides were filtered for a maximum false discovery rate of 1%. At least one unique peptide with a posterior error probability of less than 0.05 was accepted for quantification using the grouped TMT-reporter ions and proteins.

### Ingenuity pathway analysis of the vaccinated DC proteome

The Ingenuity Pathway Analysis program (IPA) is an intuitive web-based application for rapid and accurate analysis and interpretation of the biological meaning in genomic and proteomic data. Predicted protein-protein interaction networks and canonical pathways were generated from the mass spectrometer data analysis of dendritic cell proteins isolated before and after vaccine inoculation by IPA Software (Ingenuity Systems, www.ingenuity.com) (Qiagen, USA). Analysis of networks and pathways were made using log_2_ fold-changes and p-values between two-group comparisons. The ratios of significant protein expression levels were determined at r = 1.25.

### Determination of vaccinated dendritic cell IDO1 mRNA expression

Immature DCs were incubated with or without CTB-INS (10 μg/ml) and ACHP (500 nM) in culture medium for 1, 3, 6, 9, 12, 24 hours. The cells were lysed using the RNA-STAT 60 RNA isolation protocol (Tel-Test, Friendswood, TX). Indoleamine dioxygenase cDNA was synthesized from 1 to 2 μg total RNA using the SydQuanti First Strand cDNA Synthesis Kit (Syd Labs, Malden, MA) according to manufacturers’ instructions. Dendritic cell *IDO1* mRNA (NM_002164.5) was quantified relative to β-actin using the following primers: *IDO1* forward, 5’-TCTGGCCAGCTTCGAGAAAG-3’ *IDO1* reverse, 5’-AGAACTAGACGTGCAAGGCG-3’; β-actin forward, 5’-GCATTGCTTTCGTGTAAATTATGT-3’; β- actin reverse, 5’-ACCAAAAGCCTTCATACATCTCA-3’. Quantitative reverse transcriptase-polymerase chain reaction (RT-PCR) was initiated by SYBR Green JumpStart Taq ReadyMix (BioRad) according to the manufacturer’s instructions. The PCR reactions were performed in a CFX-96 BioRad C-1000 thermal cycler (BioRad Laboratories, Hercules, CA). Analysis of the data was completed with BioRad CFX manager software version 2.1 (BioRad Laboratories). All the PCR measurements were performed in triplicate and validated when the difference in threshold cycle (Ct) between the 3 measurements was < 0.3. The ratio of gene of interest/housekeeping gene was calculated according to the formula: ratio = 2^-dCt^ (dCt = mean Ct _gene_—mean Ct _housekeeping_). Dendritic Cell synthesized β-actin was used to normalize for the presence of *IDO1* mRNA. To establish statistical significance, the experiment was performed five times per subject DC collection.

### Detection of IDO1 protein synthesis in vaccinated dendritic cells

Approximately 2–9 × 10^6^ monocyte-derived DCs generated from each of several subjects were treated with CTB-INS (10 μg/ml), CTB (5μg/ml) (Sigma-Aldrich), human insulin (5μg/ml) (Sigma-Aldrich), recombinant human *E*. *coli*-derived proinsulin (5 μg/ml) (R&D Systems) and c-peptide (2 μg/ml) (Sigma-Aldrich). The inoculated DCs were lysed in buffer C (20 mM HEPES, 0.42 M KCl, 26% Glycerol, 0.1 mM EDTA, 5 mM MgCl_2,_ 0.2% NP40) containing a tablet of complete protease inhibitor (Roche, Basel, Switzerland) according to the manufacturer instruction. At least 50 μg of protein isolated from the total DC lysate was separated by electrophoresis on a 12% polyacrylamide gel (SDS-PAGE). After transfer of the separated proteins to polyvinylidene difluoride (PVDF) membranes (Millipore, Temecula, CA). The presence of IDO1 protein (NP_002155.1) was detected by incubation of the blot for 12 hours at 4^o^ C with an anti-IDO1 rabbit monoclonal primary antibody (Cat. 04–1056, clone EPR1230Y) (Millipore, Temecula, CA). For signal detection, the blot was washed 3 times with PBST and incubated for 2 hours at room temperature in the presence of a monoclonal anti-rabbit IgG γ-chain specific alkaline phosphatase conjugated secondary antibody (Cat. A-2556, clone RG-96) (Sigma-Aldrich). The blots were washed 3 times in PBST (1X PBS, 0.02% tween 20, pH 7.4) and were incubated in 200 μL of Novex AP chemiluminescent substrate (Invitrogen) for 5 minutes. The chemiluminescent labeled blot was exposed to x-ray film (Kodak X-Omat) for 3 minutes. The IDO signal intensity was quantified via Image J software v. 1.48h. (Image J, NIH)

### Flow cytometric analysis of vaccinated dendritic cells

Peripheral blood monocytes were plated in 96 well plates in RPMI 1640 culture medium containing 10% FBS and divided into two experimental groups. One group was inoculated with CTB-INS (10μg/ml) and GMCSF (50ng/ml) and IL-4 (10 ng/ml) and the second group was inoculated with GMCSF and IL-4 without CTB-INS. The treated cells were cultured for 2, 4 and 6 days. After incubation, the differentiating DCs were stained for flow cytometry with the following antibodies: anti-CD14 FITC (Miltenyi), anti-CD86 PE, anti-HLA-DR PerCP (Beckton Dickenson), anti-CD11c PE-Cy7 (Biolegend) and anti CD83 APC (Miltenyi). The differentiating cells were identified by flow cytometry (MacsQuant, Miltenyi Inc) and the data analyzed using FlowJo software v.7.6 (Treestar).

### Pharmacological Inhibitors of NF-κB activation

The NF-κB inhibitor dehydroxymethylepoxyquinomicin (DHMEQ) was provided by the laboratory of Dr. Kazuo Umezawa, Department of Molecular Target Medicine Screening, Aichi Medical University, School of Medicine, Nagakute, Aichi 480–1195, Japan. DHMEQ was shown to successfully inactivate NF-κB p50/p65, and p52/RelB subunits [30]. The DHMEQ was dissolved in DMSO at a concentration of 3.0 mg/ml and administered to CTB-INS vaccinated dendritic cell cultures at a final concentration of 3μg DHMEQ/ml culture medium. The cells were incubated in culture medium containing the inhibitor for 24 hours at 37°C.

The Iκβ kinase inhibitor, 2-Amino-6-[2-(cyclopropylmethoxy)-6-hydroxyphenyl]-4-(4-piperidinyl)-3-pyridinecarbonitrile (ACHP), (Tocris Bioscience, Bristol, UK), was dissolved in DMSO at a concentration of 25 mM and added to the culture to a final concentration of 500 nM. ACHP is a selective inhibitor for IKKα and IKKβ (IC_50_ values are 8.5 and 250 nM for IKKβ and IKKα respectively). ACHP was shown to inhibit DNA binding activity of NF-κB and to block NF-κB pathways in multiple myeloma cell lines [31].

### Determination of IDO1 enzymatic activity in vaccinated dendritic cells

Approximately 3–9 × 10^6^ monocyte-derived CD14^-^ HLADR^+^ dendritic cells from three subjects were cultured in RPMI medium + 10% fetal bovine serum in the presence or absence of CTB-INS fusion protein (10 μg/ml) at 37°C for 24 hours in 8.8 cm Nunc cell culture plates (cat. 150318) (Thermo Scientific, Waltham, MA, USA). The cells were harvested by scraping the plate with a rubber policeman. The DCs were washed twice with PBS and centrifuged at 524 × g in a Beckman Coulter Allegra X-15R centrifuge at 4°C and the pellet re-suspended in 0.5 ml of ice cold PBS. The cells were lysed by incubation in lysis buffer C containing 20 mM HEPES, pH = 7.8, 0.42 M KCl, 26% Glycerol, 0.1 mM EDTA, 5mM MgCl_2,_ 0.2% NP40 and a Roche complete protease inhibitor cocktail. The protein concentration in the supernatant was measured by Bradford assay [32] with BSA as the standard, to permit assessment of equal protein concentrations among homogenate samples and to reduce sample bias.

To measure changes in kynurenine concentrations following vaccine inoculation, the standard kynurenine assay mixture (100 μl total volume) contained 50 mM potassium phosphate buffer (pH 6.5), 20 mM ascorbate, 10μM methylene blue, 100 μg/ml of catalase and an appropriate amount of the cellular extract (10–100 μL) containing equal total protein concentration between samples. The reaction was initiated by adding the substrate L-tryptophan (400 μM) and terminated after 1 hour incubation at 37°C. A volume of 20 μL of 30% trichloroacetic acid was added to the mixture followed by incubation of the mixture at 50°C for 30 minutes to terminate the reaction and to hydrolyze the N-formyl kynurenine cleaved by IDO1 to L-kynurenine [33]. After centrifugation, the supernatants were divided into 2 experimental groups. The first group was treated with 100 μM final concentration of the IDO1 inhibitor L-1-methyl-tryptophan (L-1-MT) and the other was left untreated.

The optical density of the samples was measured at 490 nm. The absorbance values were plotted against a standard curve of defined kynurenine amounts (0–100 μM). Kynurenine determinations were repeated 3 times for each subject from a total of 3 subjects. After comparison with the standard curve of kynurenine concentrations, the data were presented as IDO1 activity. One unit of the enzyme activity was defined as the amount of enzyme that produced 1 nmol of kynurenine/h.

### Statistical analysis

All the quantitative data of this study were expressed as the mean ± SD and statistical analysis was conducted using GraphPad Prism software v.6.01 (La Jolla, CA). Comparisons between appropriate groups were performed with paired Student’s t-test and probability p <0.05 was considered to indicate a statistically significant difference. Each experiment was repeated at least twice to assess the level of reproducibility.

### Monocyte and dendritic cell viability in the presence of IDO1 inhibitors

Monocyte derived dendritic cells were treated with ACHP (500 nM) and DHMEQ (3 μg/ml) for 24 hours and stained with annexin V and propidium iodide (PI) to determine cell viability. The percentage of DCs negative for annexin V and PI was obtained by flow cytometry analysis.

## Results

### CTB-INS modulates the dendritic cell proteome

**Fig 1:**
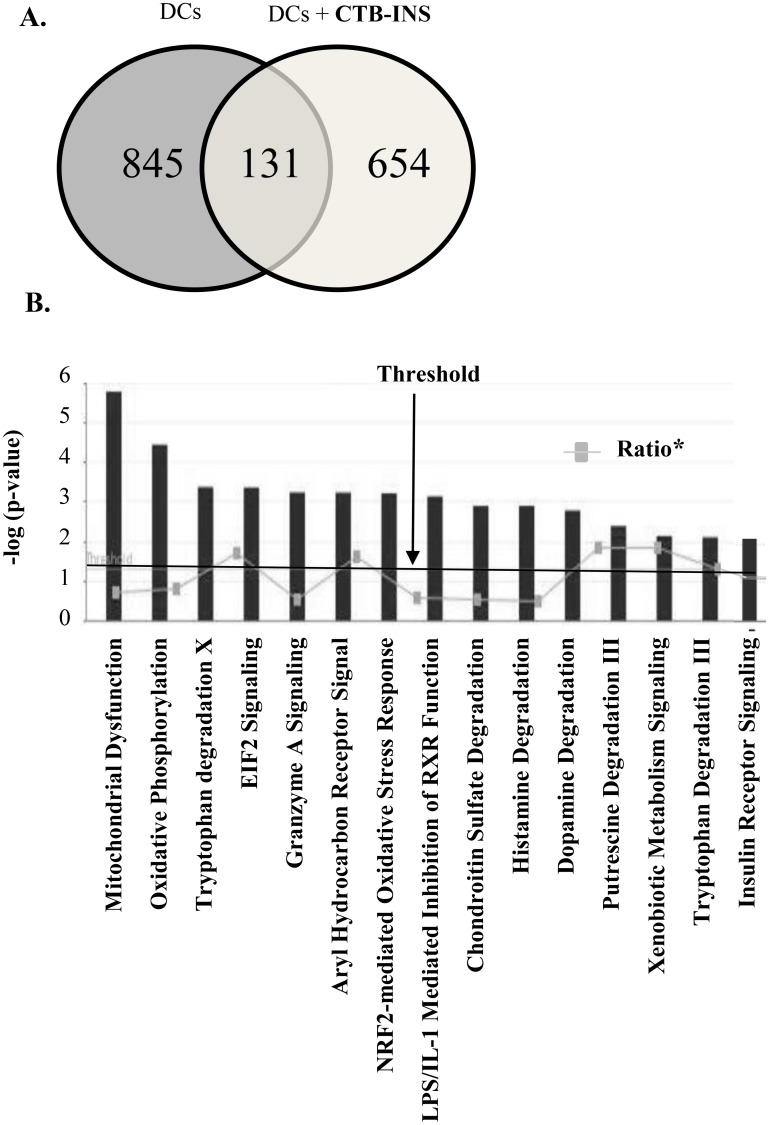
Mass spectrometric identification of the CTB-INS vaccinated DC proteome. (A) Venn diagram of significantly up or down regulated proteins in human monocyte derived dendritic cells inoculated with CTB-INS in comparison with monocytes as the baseline. A total of 845 proteins were identified in untreated DCs and 654 up and down-regulated proteins were unique in CTB-INS treated DCs, while 131 proteins were common to both treated and untreated DCs. (B) Canonical, metabolic and signaling pathways that correlate with changes in protein expression in CTB-INS vaccinated dendritic cells identified by IPA program analysis. The threshold line represents the value at which statistical significance is reported. The grey line connects the ratios between the number of up and down-regulated proteins over the total number of proteins identified in each of the pathways shown.

Dendritic cells were both treated with 10μg/ml of CTB-INS or PBS for 24 hours and lysed. To determine the effects of CTB-INS vaccination on the DC proteome, we employed Orbitrap mass spectrometry to identify and quantify vaccine regulated proteins and Ingenuity Pathway Analysis (IPA) algorithms, to assess their role in signaling or metabolic functions in vaccinated DCs. A total of 845 proteins were identified in unvaccinated DCs and 654 proteins were identified as unique and significantly up or down regulated in CTB-INS vaccinated DCs. About 131 proteins were detected as common in both vaccinated and unvaccinated DCs (Fig. 1A). Signaling pathways identified by IPA that correlated with changes in protein expression in vaccinated DCs were arranged by decreasing—log (p-values) (Fig. 1B). Identified among these pathways were 2 types of tryptophan degradation pathways, namely the tryptophan degradation pathways III and X [34]. Ingenuity pathway analysis of the ratios of up and down-regulated proteins over the total number of proteins in each DC metabolic or signaling pathway showed that the tryptophan degradation pathway, the aryl hydrocarbon receptor signaling pathway and the dopamine degradation pathway were significantly up-regulated in the presence of the vaccine (Fig. 1B).

**Fig 2:**
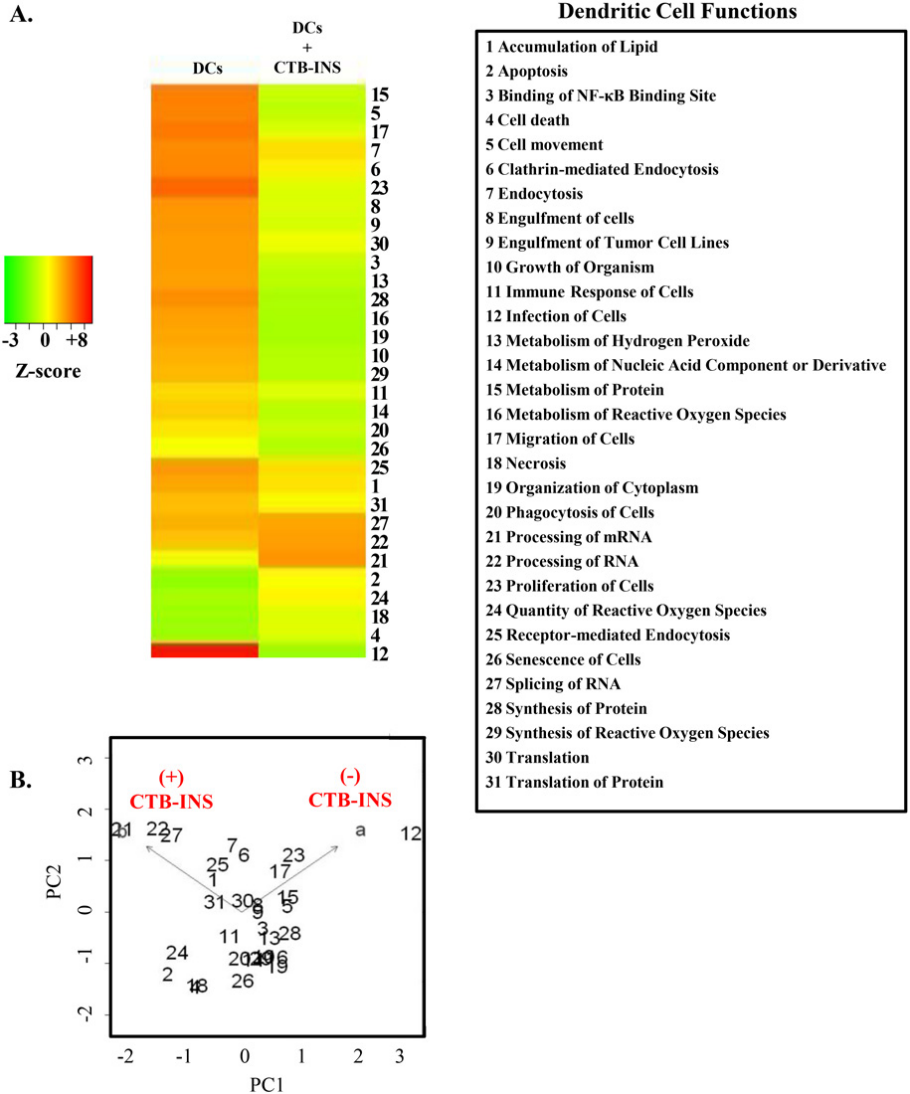
CTB-INS vaccine induced changes in DC cellular functions. (A) Heat map representing cellular functions (z-scores*) identified in vaccinated dendritic cells. Red bands indicate up-regulated cellular functions, yellow bands indicate no change in function and green bands indicate down-regulated cellular functions. (B) Principal component (PC) analysis of dendritic cell functions, z-scores. The Bi-plot correlates dendritic cell functions (+/-) vaccine treatments as indicated by the proximity of individual DC functions (numbers) to either vector arrowhead [a = CTB-INS (-), b = CTB-INS (+)]. Numbers further from the mean in the direction of the vector exhibit an increased correlation of the DC function with a or b. Example: Function #12 is highly correlated with unvaccinated DCs, [CTB-INS (-)] and Function #2 is poorly correlated with vaccinated DCs, [CTB-INS (+)]. Zero (0) is the weighted mean of the multivariate dataset. The values on the (x; y) axis represent (+/-) deviation from the mean of the dataset (0). (*z-score = statistical measurement of a score’s relationship to the mean in a group of scores. A z-score of 0 means the score is the same as the mean. A z-score can also be positive or negative, indicating whether it is above or below the mean and by how many standard deviations.)

The effect of CTB-INS vaccination on changes in DC cellular functions including protein synthesis, maturation, and the immune response are presented graphically in the heat map (Fig. 2A). Dendritic cell functions (shown as z-scores), are displayed as up-regulated (red bands), down-regulated (green bands) or insignificantly changed (yellow bands), following DC inoculation with the vaccine. Dendritic cell functions identified by IPA that correlate with bands in the heat map are labeled as numbers to the right of the map. Functions associated with mechanisms leading to increased dendritic cell maturation, such as the immune responses of dendritic cells, cell movement, and cell migration, were found to be significantly down-regulated in CTB-INS treated dendritic cells [35–37]. Integrin associated protein (CD47), involved in a wide range of inflammatory cellular processes including activation, proliferation, migration and immune responses [38–40], was also found to be markedly down-regulated (Table 1). The dataset used to generate the heat map was analyzed by principal component (PC) analysis in order to identify the extent of cellular function correlation with CTB-INS treatment (Fig. 2B). As predicted from the heat map, CTB-INS vaccination induced distinct cellular functions in DCs that correlated with a change toward immune suppression which were absent from unvaccinated DCs. Cellular functions up-regulated in CTB-INS (+) treated DCs include RNA splicing and processing of mRNA which appear on the plot near the arrowhead of the CTB-INS (+) vector, indicating a high level of correlation with DC vaccination. Alternatively, pro-inflammatory cellular functions such as infection of cells, are strongly down-regulated in CTB-INS (+) DCs, and are located near the arrowhead of the CTB-INS (-) vector.

**Table 1:** Effect of CTB-INS on dendritic cell proteins associated with maturation and activation.

↓Dendritic Cell Maturation/Activation		
Molecule	Fold Change	Function
**IDO1 (Indoleamine 2,3-dioxygenase 1)**	**↑**1.56	An immune-modulatory enzyme that catalyzes the degradation of the essential amino acid L-tryptophan to N-formylkynurenine. Also known as a marker of tolerogenic dendritic cells [44,57].
**CD86**	**↓**1.58	Type I membrane protein that is a member of the immunoglobulin superfamily which provides co-stimulatory signals necessary for T cell activation and survival [64].
**TYROBP** (TYRO protein tyrosine kinase binding protein)	**↓**1.98	A transmembrane signaling polypeptide which contains an immune-receptor tyrosine-based activation motif (ITAM) in its cytoplasmic domain associated with inflammation [44,65].
**CD47**	**↓**2.07	Transmembrane protein belonging to the immunoglobulin superfamily. Involved in migration and efficient initiation of the immune response [38–40].
**BTK** (Bruton agammaglobulinemia tyrosine kinase)	**↑**1.53	A kinase mediating TLR9 responses in dendritic cells [66].
**CD209** (Dendritic Cell-Specific Intercellular adhesion molecule-3-Grabbing Non-integrin)	**↑**1.57	C-type lectin receptor which mediates dendritic cell rolling and activation of CD4^+^ T cells, as well as recognition of pathogen haptens [67].
**MPO** (myeloperoxidase)	**↓**1.85	An enzyme that produces hypochlorous acid from hydrogen peroxide and chloride anion and oxidizes tyrosine to tyrosyl radical. Dendritic cells use hypochlorous acid and tyrosyl to kill pathogens in innate immunity [68].

### CTB-INS vaccination induces IDO1 biosynthesis in human dendritic cells

To address how CTB-INS interacts with DCs to suppress T1D, we used the IPA program to identify up or down-regulated DC proteins that might be associated with DC activation (maturation) and the onset of T1D (Tables 1 and 2). Among these proteins, indoleamine 2, 3-dioxygenase (IDO1), the first enzyme of the tryptophan degradation pathway, was found to be significantly up-regulated.

**Table 2:** Effect of CTB-INS on proteins associated with type 1 diabetes onset.

↓Type 1 Diabetes		
Molecule	Fold Change	Function
**CD86**	**↓**1.58	(See Table 1). Associated with development of T1D [69].
**HIST2H2BE** (histone cluster 2, H2be, includes others)	**↓**1.66	Nuclear proteins responsible for the nucleosome structure of the chromosomal fiber in eukaryotes. Single nucleotide polymorphism (substitution mutation, allelic variations: A/G (rs806792) is associated with T1D humans [70].
**TYROBP** (TYRO protein tyrosine kinase binding protein)	**↓**1.98	Up-regulation of mouse TYROBP mRNA in islets of Langerhans is associated with type I diabetes in NOD mouse and humans [71].
**SGCZ** (sarcoglycan, zeta)	**↓**2.35	N-glycosylated transmembrane proteins which are part of the dystrophin-associated glycoprotein complex. This complex connects inner cytoskeleton to extracellular matrix. Single nucelotide polymorphism (mutation, allelic variations: C/G (rs1365641) is associated with insulin-dependent diabetes mellitus in humans [70].
**IDO1 (Indoleamine 2,3-dioxygenase 1)**	**↑**1.56	(See Table 1) Defective expression in NOD mice [43]

Dendritic cells were incubated in the presence or absence of CTB-INS for 24 hours. *Orbitrap* mass spectrometry was used to identify the DC proteins and their relative abundance. Molecules associated with the up or down- regulation of dendritic cell maturation were identified by IPA program analysis. The up and down arrows indicate proteins significantly up or down-regulated during CTB-INS suppression of DC maturation and activation.

Dendritic cells were incubated in the presence or absence of CTB-INS for 24 hrs. *Orbitrap* mass spectrometry was used to identify DC proteins and their relative abundance. Molecules associated with the onset of T1D were identified by IPA program analysis. The arrows indicate significant down-regulation of proteins involved in insulin-dependent diabetes (T1D) onset by treatment with the CTB-INS vaccine.

**Fig 3:**
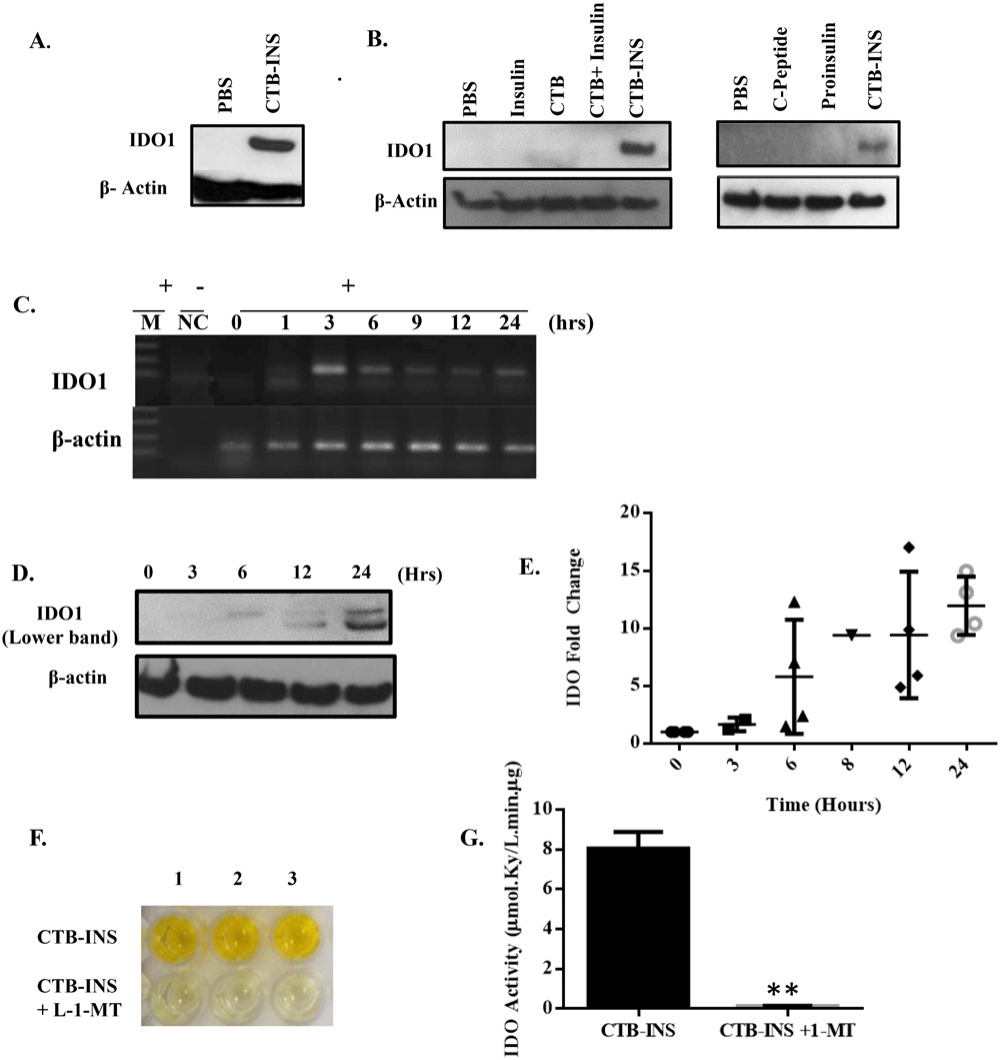
Vaccine induction of IDO1 biosynthesis in dendritic cells. (A) Immunoblot detection of IDO1 protein synthesis in human DCs in the presence of CTB-INS. **(B)** Immunoblot analysis of IDO1 up-regulation in DCs inoculated with all components of the CTB-INS fusion protein including insulin C-peptide. The images are representative of five subjects. **(C)** Reverse transcriptase polymerase chain reaction (RT-PCR) for assessment of *IDO1* mRNA expression in a time dependent manner from DCs incubated with and without CTB-INS. RNA (2 μg). The cDNA samples were used as templates for PCR amplification. The DNA amplification products were visualized on X ray film after electrophoresis in 2.0% agarose gels. *Lane M*: 100 bps DNA ladder marker (*Gibco*); *lane NC*: negative control RNA after treatment with DNase I from DCs as template; *lane 0*: cDNA from DCs without CTB-INS inoculation (-); *lanes 1*, *3*, *6*, *9*, *12 and 24*: cDNA from time-dependent DCs incubated with CTB-INS (+). A β-actin gene was used as an internal standard in RT-PCR.The image is representative of 3 different subjects. **(D, E)** Immunoblot of a time course of IDO1 expression in DCs after inoculation with CTB-INS (10μg/mL). Cell samples were harvested at 0, 3, 6, 8, 12 and 24 hours after vaccine inoculation. The data is representative of 6 different subject samples. (Shapes on Panel E represent different subjects). **(F, G)** Assessment of vaccine induced IDO1 activity and L-1-methyl-L-tryptophan (L-1-MT) inhibition of IDO1 activity in vaccinated DCs identified by ELISA. Top wells in the plate (F), show the production of tryptophan degradation products (kynurenines) in vaccinated DCs. Bottom wells show the inhibition of IDO1 kynurenine biosynthesis (absence of yellow color), in vaccinated DCs treated with L-1-MT (bottom wells). The data was compared for significance using a paired t-test, p = 0.018.

To confirm the mass spectrometry data, we assessed CTB-INS induced up-regulation of IDO1 in DCs by immunoblotting and found that DCs treated for 24 hours with CTB-INS (10μg/ml) showed a significant increase in IDO1 biosynthesis (Fig. 3A). To determine the specificity of CTB-INS fusion protein for induction of IDO1, dendritic cells were incubated with CTB, insulin, proinsulin, c-peptide, a combination of both CTB and insulin, and CTB-INS fusion protein for 24 hours followed by immunoblot analysis. Unlike CTB-INS, none of the vaccine components delivered alone or together induced IDO1 biosynthesis (Fig. 3B). The CTB-INS fusion protein induced detectable levels of IDO1 in DCs as early as 3–6 hours after treatment and *IDO1* RNA levels as early as 3 hours. (Fig. 3C-E). These results clearly demonstrated CTB-INS-induced IDO1 is enzymatically active in conversion of L-tryptophan into L-kynurenine. Kynurenine biosynthesis was arrested by 100 μM of the IDO1 inhibitor L-1-methyl-tryptophan (1-MT) as shown in Fig. 3F-G.

### NF-κB inhibitors ACHP and DHMEQ block CTB-INS vaccine induced IDO1 biosynthesis

**Fig 4:**
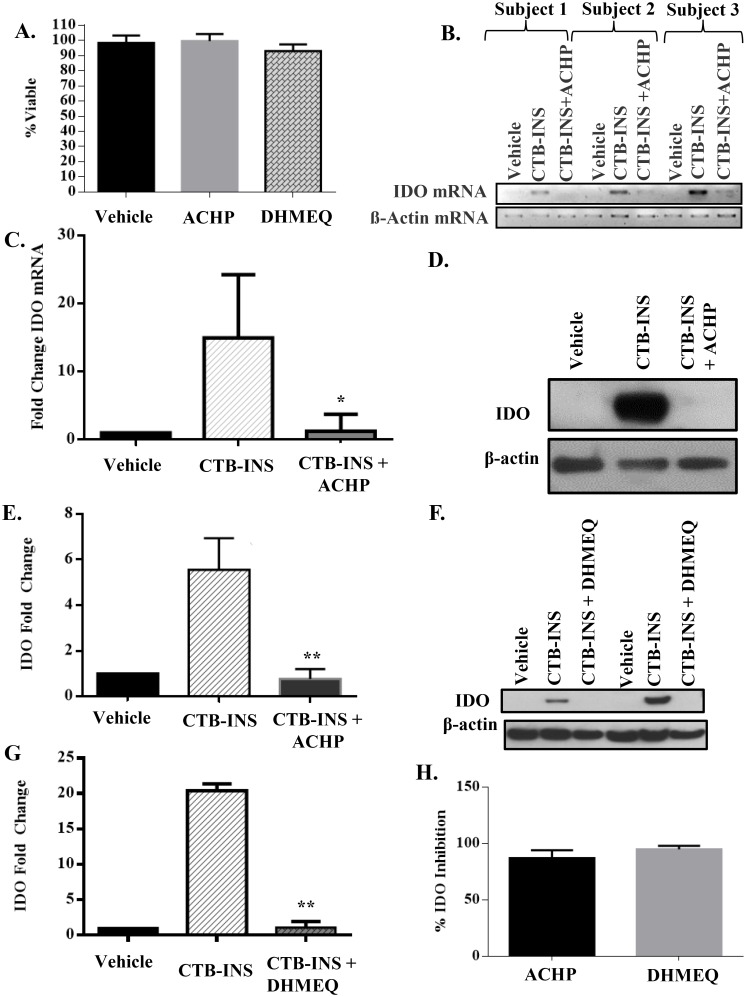
ACHP and DHMEQ inhibitors of NF-κB block CTB-INS induced IDO1 biosynthesis. **(A)** Dendritic cell viability was measured by determination of the percentage of DCs negative for annexin V and propidium iodide following treatment with ACHP (500 nM) and DHMEQ (3μg/ml). The measurement of DC viability shows vaccinated DCs are unaffected by treatment with the inhibitors. **(B, C)** Agarose gel electrophoresis and graphic representation showing changes in *IDO1* mRNA levels in DCs from three representative subjects determined by qRT-PCR after 3 hours incubation of subject monocyte derived DCs with CTB-INS (10 μg/mL) or CTB-INS and ACHP. Statistical significance (p<0.05) was achieved by analysis of a total of 5 subjects. (The vehicle used is the DMSO solvent in which ACHP and DHMEQ are dissolved). **(D, E)** Immunoblot and graphic representation of the fold change in IDO protein synthesized 24hrs after vaccine inoculation of subjects DCs inoculated with CTB-INS +/- ACHP. The experimental data was collected from five normal subjects, although only one representative subject is shown. Statistical significance between vaccine treated and vaccine + ACHP treated groups was measured by paired Student’s t test, (p = 0.0273). **(F, G)** Immunoblot and graphic representation of the fold change in IDO1 protein synthesized in subject DCs following inoculation with CTB-INS with and without DHMEQ. While experimental data was collected from five normal subjects, only two are shown to highlight the variability in IDO1 levels observed among subjects. Statistical significance was measured by paired Student’s t test (p = 0.017). **(H)** Graphic representation showing the relative efficacy of ACHP and DHMEQ inhibitors for blocking NF-κB induction of IDO1 biosynthesis.

Based on the observation that CD40 ligand (CD40L) induces IDO1 biosynthesis through NF-κB signaling in human DCs [41], we investigated the role of NF-κB signaling pathway involvement in CTB-INS induced IDO1 biosynthesis. To assess the requirement for NF-κB in vaccine up-regulation of IDO1 we attempted to block vaccine stimulated IDO1 biosynthesis in DCs with two different NF-κB pharmacological inhibitors, ACHP and DHMEQ. Assessment of DC viability following ACHP and DHMEQ treatment indicated both inhibitors were not toxic to DCs (Fig. 4A). Immature dendritic cells were incubated with CTB-INS +/- the NF-κB inhibitor ACHP. The levels of *IDO1* mRNA were found to be significantly increased in vaccinated DCs while addition of ACHP to vaccinated DCs completely inhibited IDO1 transcription (Fig. 4B-C). Correspondingly, IDO1 protein levels were significantly higher in cells inoculated with CTB-INS than in DCs incubated for 24 hours with CTB-INS + either ACHP or DHMEQ (Fig. 4D-G). Both DHMEQ and ACHP were shown to be equally effective in blocking DC biosynthesis of IDO1 (Fig. 4H).

**Fig 5:**
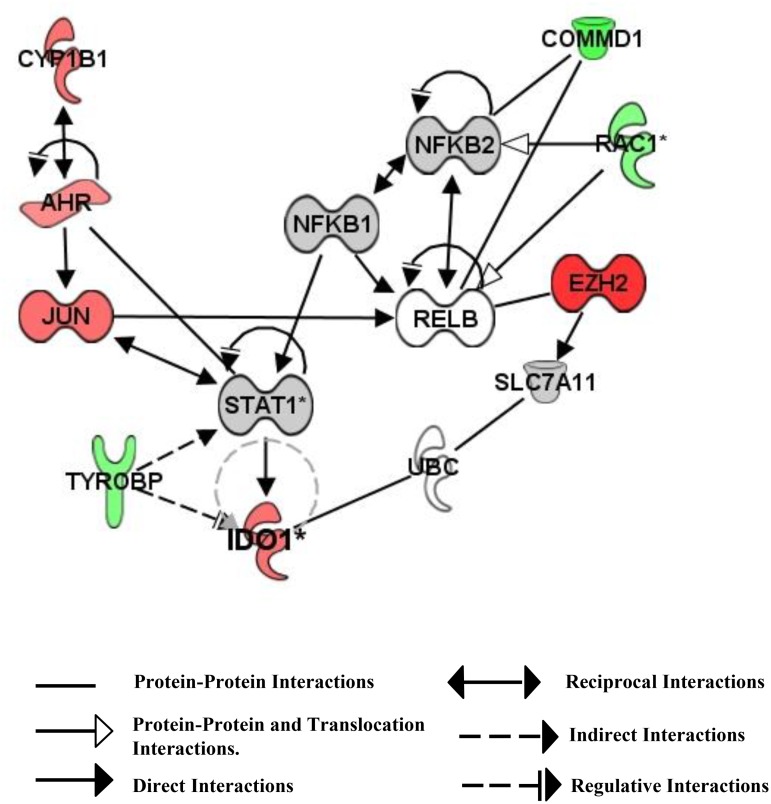
Diagram of upstream signals that regulate IDO1 biosynthesis in CTB-INS vaccinated human DCs. Signaling pathways involved in vaccine up-regulation of IDO1 biosynthesis in human DCs were identified by Ingenuity Pathway analysis of proteins detected in vaccinated DCs by mass spectrometer analysis. (Red: up-regulation of the protein, Green: down-regulation of the protein, Grey: protein levels unchanged, White: Undetected). Based on calculations generated from the mass spectrometry data, the IPA program indicates the vaccine up-regulates the aryl hydrocarbon receptor (AhR) signal transduction pathways and down regulates TYROBP to increase IDO biosynthesis. **NF-κB1**: is the P105 precursor subunit of NF-κB which is cleaved to generate the P50 subunit of the canonical NF-κB transcriptional activator protein, located in the cytoplasm. **NF-κB2**: is the P100 subunit of non-canonical NF-κB, cleaved to generate the p52 subunit, located in the cytoplasm.

The mass spectrometry proteome data from vaccine treated DCs was compared with a network of upstream regulators of IDO1 identified by IPA. The experimental results indicated that vaccine induced expression of *IDO1* mRNA may be dependent not only on NF-κB, but also on AhR and STAT1 transcriptional activators as well as vaccine-induced down-regulation of the tyrosine kinase binding protein (TYROBP) [42–44] (Fig. 5).

**CYP1B1**: Cytochrome P450, Family 1, Subfamily B, polypeptide 1, cell location-Cytoplasm (up-regulated, 1.540 fold). **AhR**: Aryl Hydrocarbon Receptor, located in the cytosol and nucleus, (up-regulated, 1.270 fold). **COMMD1**: Copper metabolism (Murr-1) domain containing 1, located in cytosol and nucleus, (down-regulated 2.748 fold). **RAC-1**: ras-related C3 botulinum toxin substrate 1. Located in the inner plasma membrane, (down-regulated 1,895 fold.) **JUN**: JUN proto-oncogene, location, nucleus, (up-regulated, 1.584 fold.) **EZH2**: Enhancer of Zeste homolog 2, a histone methyltransferase that forms part of the polycomb repressive complex-2, located in the nucleus, (up-regulated 7.244 fold). **TYROBP**: Tyrosine kinase binding protein, located in the plasma membrane (down-regulated 1.982 fold). **IDO1**: Indoleamine 2, 3 dioxygenase, located in the cytoplasm, (up-regulated 1.556 fold). Fold regulation values presented represent the average of 7 subjects.

### CTB-INS does not affect monocyte differentiation into dendritic cells

**Fig 6:**
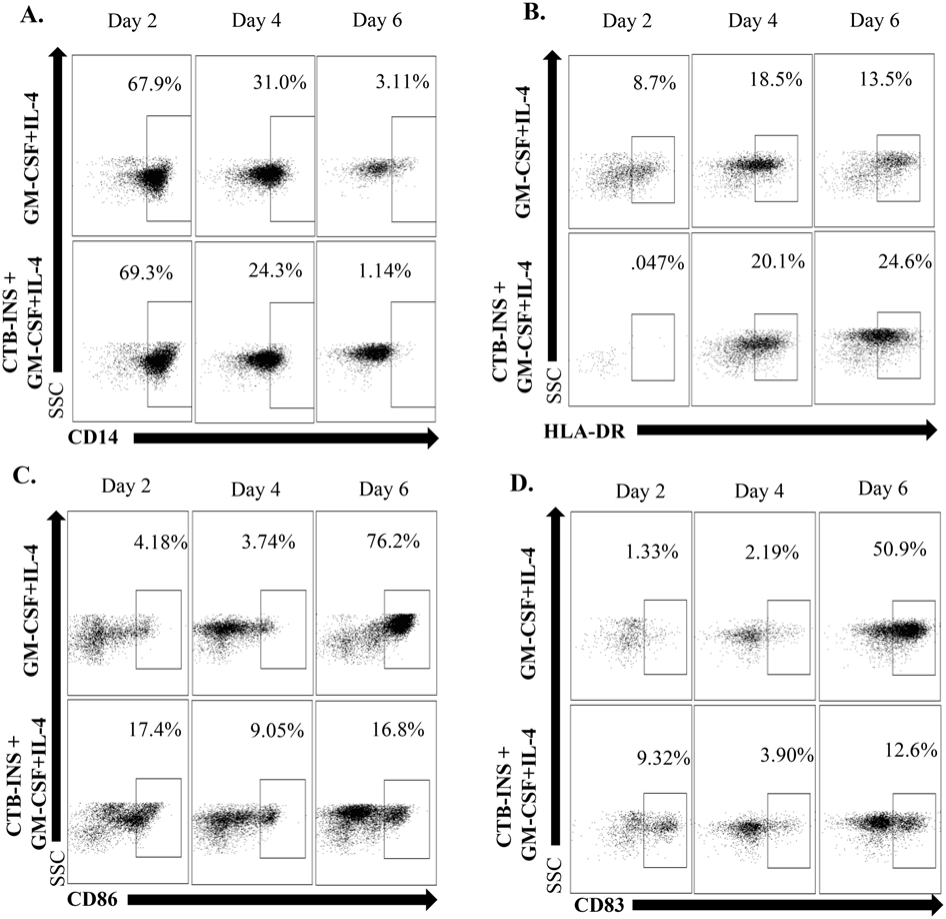
CTB-INS vaccination of monocytes does not interfere with dendritic cell differentiation. Flow cytometric analysis of vaccinated monocyte differentiation into dendritic cells. **(A)** The CD14 monocyte marker becomes progressively less positive (number inside the box (gate), as DC differentiation proceeds in the presence of the CTB-INS fusion protein. **(B)** The HLA-DR marker indicative of antigen presenting cell DC morphogenesis increases as dendritic cell differentiation progresses in the presence of CTB-INS. **(C)** The co-stimulatory factor marker for DC maturation CD86 is observed to decrease with time in vaccinated DCs in comparison with background levels. **(D)** With time after inoculation with CTB-INS, the dendritic cell marker CD83 indicative of DC maturation decreases in comparison with unvaccinated DC background levels.

Despite phenotypic and functional differences based on their origin, maturation stage, and culture conditions, DCs display common features among their subsets. These features include similar morphology, high density of membrane HLA class II co-stimulatory molecules, low phagocytic activity, and a strong capacity for antigen uptake and presentation to T lymphocytes [45,46]. Under normal conditions of immunological homeostasis, monocytes are more numerous than DCs and can differentiate into dendritic cells [47]. To assess vaccine safety for clinical applications, we examined potential detrimental effects of vaccine inoculation on the differentiation of monocytes into dendritic cells (Fig. 6). Peripheral blood monocytes were isolated and inoculated with CTB-INS plus GM-CSF and IL-4 in one experimental group and another group of monocytes were inoculated with only GM-CSF and IL-4 only. Both experimental groups were cultured for 2, 4 and 6 days post inoculation. The disappearance of the CD14 monocyte markers and appearance of the dendritic cell MHC Class II HLA-DR markers were monitored during DC differentiation. The morphogenesis of monocytes into dendritic cells was not impeded by inoculation with CTB-INS and the levels of DC maturation markers CD86 and CD83 measured by flow cytometry were lower than the controls in monocyte cultures inoculated with CTB-INS (Fig. 6C-D). The time required for differentiation from monocytes to DCs was not altered by inoculation with CTB-INS. In addition, DCs treated with the vaccine showed a modulation of proteins involved in the suppression of DC maturation and activation (Table 1) along with down regulation of many proteins involved in the onset of type 1 diabetes (Table 2).

## Discussion

Our laboratory and others have previously demonstrated the phenomenon of suppression of insulitis and autoimmune diabetes in CTB-INS vaccinated NOD mice [14,15,28]. However, the cellular and molecular mechanisms underlying CTB-INS modulation of dendritic cell mediated immune suppression of T1D remain poorly understood. Because DCs are arguably the most important population of antigen presenting cells involved in the induction of inflammatory and auto-reactive T cell morphogenesis, we investigated the effects of CTB-INS on human immature dendritic cell activation (maturation) *ex vivo* and attempted to correlate the experimental data with findings obtained from earlier NOD mice studies.

Analysis of the mass spectrometry data by IPA algorithms indicated that a significant number of dendritic cell proteins (654) were uniquely modulated either negatively or positively by vaccination with CTB-INS (Fig. 1A). Metabolic or signaling pathways including two tryptophan degradation pathways (IDO, INDO), the aryl hydrocarbon receptor (AhR) and the dopamine degradation pathway (DA), demonstrated a significantly high ratio of vaccine modulated proteins (Fig. 1B).

Dendritic cells inoculated with the CTB-INS fusion protein demonstrated a general reduction in their metabolism and immune functions (Fig. 2). Dendritic cell functions identified by IPA that showed a high number of pro-inflammatory functions involved with DC activity, movement and migration were down-regulated by the vaccine. Inflammatory functions such as infection, migration and proliferation of dendritic cells (Fig. 2B, Functions **12, 17**, and **23**) were found near the arrowhead of the CTB-INS (-) vector. These results suggest CTB-INS may exert an anti-inflammatory function on DCs. Alternatively, dendritic cell functions shown to be up-regulated such as RNA splicing and processing of mRNA (Fig. 2B, functions **21**, **22** and **27**) were located near the arrowhead of the CTB-INS (+) vector on the bi-plot (Fig. 2B), suggesting a high level of *IDO1* mRNA processing induced by the vaccine. Together, these experimental findings suggest the vaccine induces changes in dendritic cell functions that favor maintenance of immunological tolerance.

Encoded by the *IDO1* gene, indoleamine 2,3-dioxygenase is the rate-limiting catabolic enzyme responsible for initiating the degradation of L-tryptophan (L-Trp) to N-formyl kynurenine and its downstream degradation products [48]. Additional experimental findings showed that in addition to tryptophan starvation of DCs, paracrine effects of secreted kynurenine tryptophan degradation products may contribute to DC tolerogenesis through initiation of inflammatory T cell apoptosis [34,49,50]. Therefore, part of the mechanism responsible for IDO1 stimulated immune suppression may reside in the inhibitory or stimulatory effects of kynurenines on T cell, regulatory T cell (Treg) and natural killer (NK) cell proliferation [51–55]. Further, both depletion of dendritic cell tryptophan coupled with the immunosuppressive action of kynurenine degradation products on T cell functions, including activation of NF-κB and additional transcription factors such as AhR, may contribute to the broad and powerful immunosuppressive actions of IDO1 [49,50]. It has recently been shown that the immunosuppressive effect of IDO1 requires both its enzymatic and signaling functions in NOD mice [56,57]. In these studies conducted by Grohmann et al., IDO1 signaling activity was triggered in plasmacytoid dendritic cells (pDCs) by transforming growth factor-β (TGF-β), through the non-canonical NF-κB pathway to induce long-lasting IDO1 expression and autocrine TGF-β production in a positive feedback loop. Additionally, IDO1 expression and catalytic function were found defective in pDCs from NOD mice. Our experimental data shows that CTB-INS induced IDO1 biosynthesis in DCs results in the production of L-kynurenine (Fig. 3). Supporting these experimental results, dendritic cells with high IDO1 expression levels were shown to generate increased tryptophan catabolites such as L-kynurenine, 3-hydroxykynurenine and 3-hydroxyanthranilic acid and were able to suppress allogeneic T-cell proliferation irreversibly *in vitro* [51,58]. Other laboratories showed that kynurenines increased the recruitment of regulatory T-cells (Tregs) [53,59].

To determine the specificity of CTB-INS fusion protein for stimulating IDO1 biosynthesis, it was necessary to test the fusion protein components for their ability to up-regulate IDO1 in monocyte-derived DCs. The lack in the induction of IDO1 expression by CTB corroborated the previous findings of Slavica et al. [60]. Interestingly, both insulin, proinsulin and the linking peptide (c-peptide) were unable to induce detectable levels of IDO1, suggesting that up-regulation of IDO1 biosynthesis is dependent on the fusion of CTB to the auto-antigen.

In addition to showing that CTB-INS increases *IDO1* mRNA and protein levels (Fig. 4A), we investigated the nature of signaling pathways required for CTB-INS induction of IDO1. Inhibition of IDO1 biosynthesis by ACHP and DHMEQ suggests that CTB-INS induction of IDO1 occurs through blocking the canonical and/or the non-canonical NF-κB signaling pathways (Fig. 4). The specific involvement of either the canonical or non-canonical NF-κB pathways in CTB-INS induction of IDO1 has yet to be elucidated. Ingenuity pathway analysis of the mass spectrometry data from vaccine treated DCs in comparison with a network of upstream regulators of IDO1 revealed the aryl hydrocarbon receptor (AhR) transcriptional activator protein to be a potential alternative mechanism that might cooperate with NF-κB for up-regulation of IDO1 (Fig. 5). Recently AhR was shown to be required for induction of IDO1 biosynthesis [61]. More recently RelB was shown to bind to AhR suggesting a cooperative effect in IDO1 up-regulation by the two transcriptional activators [62]. Additionally, N-formyl kynurenine, the first breakdown product in the IDO1-dependent tryptophan degradation pathway was shown to activate AhR leading to AhR-dependent Treg generation [55]. While it is possible there is a cooperative involvement of both NF-κB and AhR signaling pathways in CTB-INS induction of IDO1, the specific contribution of p50-RelA and p52-RelB dimers in AhR signaling during CTB-INS stimulation of IDO1 remains to be investigated.

Demonstrating the immunosuppressive function of CTB-INS, the tyrosine kinase binding protein (TYROBP) is involved in the down-regulation of IDO1 biosynthesis and was found to be markedly suppressed by the vaccine (Fig. 5, Table 1) [44]. This finding suggests a portion of the tolerogenic function of the CTB-INS vaccine may be centered on promotion as well as prevention of IDO1 down-regulation. Additionally, dendritic cell generated dopamine (DA) was shown to interact with the dopamine related protein 5 (DAR5) to produce the pro-inflammatory cytokine IL-23 in response to LPS stimulation thereby enhancing Th17 pro-inflammatory responses [63]. Ingenuity Pathway analysis indicated that CTB-INS vaccination may down-regulate DC dopamine production through increased activation of the dopamine degradation pathway (Fig. 1B).

Strengthening the opportunity for clinical testing in patients, our data showed that CTB-INS did not interfere with monocyte differentiation into DCs. This result was indicated by disappearance of the monocyte CD14^+^ marker following incubation with the vaccine (Fig. 6). In addition, dendritic cell expression of HLA-DR markers remained unaffected. Dendritic Cell maturation markers such as CD86 and CD83 are synthesized at much lower levels than normal in vaccine inoculated DCs confirming their regulatory role in vaccine induced DC tolerogenesis. Showing their potential linkage, down-regulation of co-stimulatory factors CD86 and CD83 in vaccinated DCs occurs simultaneously with IDO1 up-regulation. These experimental results correlate with CTB-INS induced suppression of DC maturation. Taken together, the data support further consideration of CTB-INS for development as a safe and effective immunosuppressive therapeutic strategy for prevention and reversal of tissue-specific autoimmunity.

## Conclusions

Our experimental data suggest CTB-INS vaccine-induced IDO1 biosynthesis is a likely mechanism for safe and effective immune suppression of DC maturation leading to the induction of durable peripheral tolerance. The mechanism for DC mediated tolerance may occur through starvation of the DC for essential amino acids by depletion of cellular tryptophan levels, the production of kynurenines known to be toxic to pro-inflammatory T cells and the induction of regulatory T cells. Understanding how CTB-INS modulates IDO1 activity in human dendritic cells will facilitate the improvement of vaccine efficacy and safety, moving this effective immunosuppressive strategy closer to clinical applications for prevention of T1D.

## Supporting Information

S1 file Immunoblot films showing results of control experiment of CTB-INS specificity of induction of IDO1 in 3 representative subjects. (JPG)

S2 file Immunoblot films showing induction of IDO1 by CTB-INS in Human dendritic cells in 2 subjects. (JPG)

S3 file Immunoblot films showing time course experiment results of IDO1 induction by CTB-INS in DCs in 2 subjects. (JPG)

S4 file Immunoblot film showing result of specificity CTB-INS fusion protein in induction of IDO1 and not part of its components. Three subjects represented.
(JPG)

S5 file Immunoblot film showing inhibition of CTB-INS-induced IDO1 by DHMEQ. Two subjects shown.
(JPG)

S6 file Immunoblot film showing inhibition of CTB-INS-induced IDO1 by ACHP. Three subjects shown.
(JPG)
