# Supplementary figures and images for: Induction of Indoleamine 2, 3-Dioxygenase in Human Dendritic Cells by a Cholera Toxin B Subunit—Proinsulin Vaccine
Source: PLoS One. 2015 Feb 25;10(2):e0118562. doi: 10.1371/journal.pone.0118562 (PMC4340906; doi:10.1371/journal.pone.0118562)

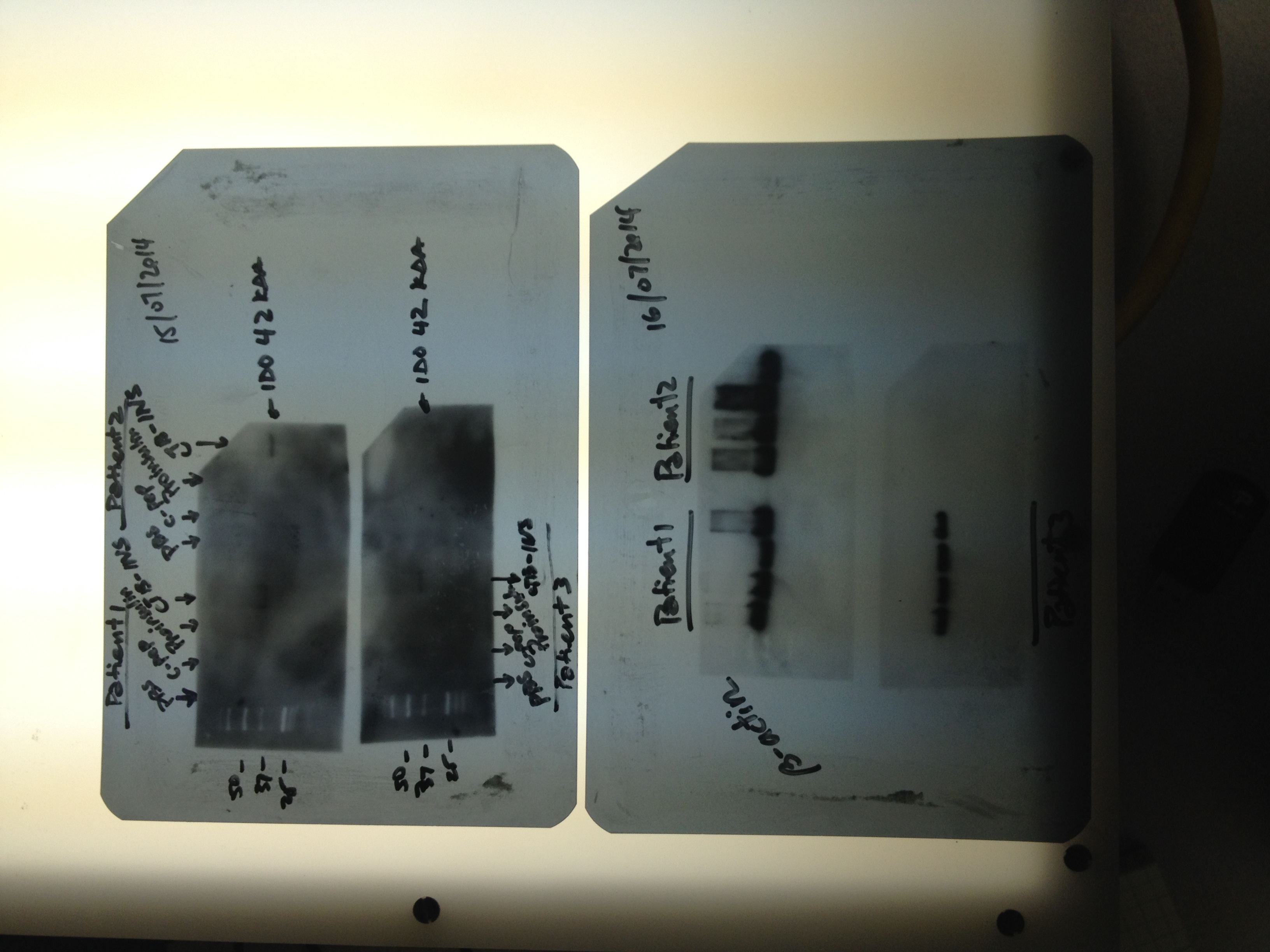

Supplement: S1 file — (JPG) [file pone.0118562.s001.JPG]

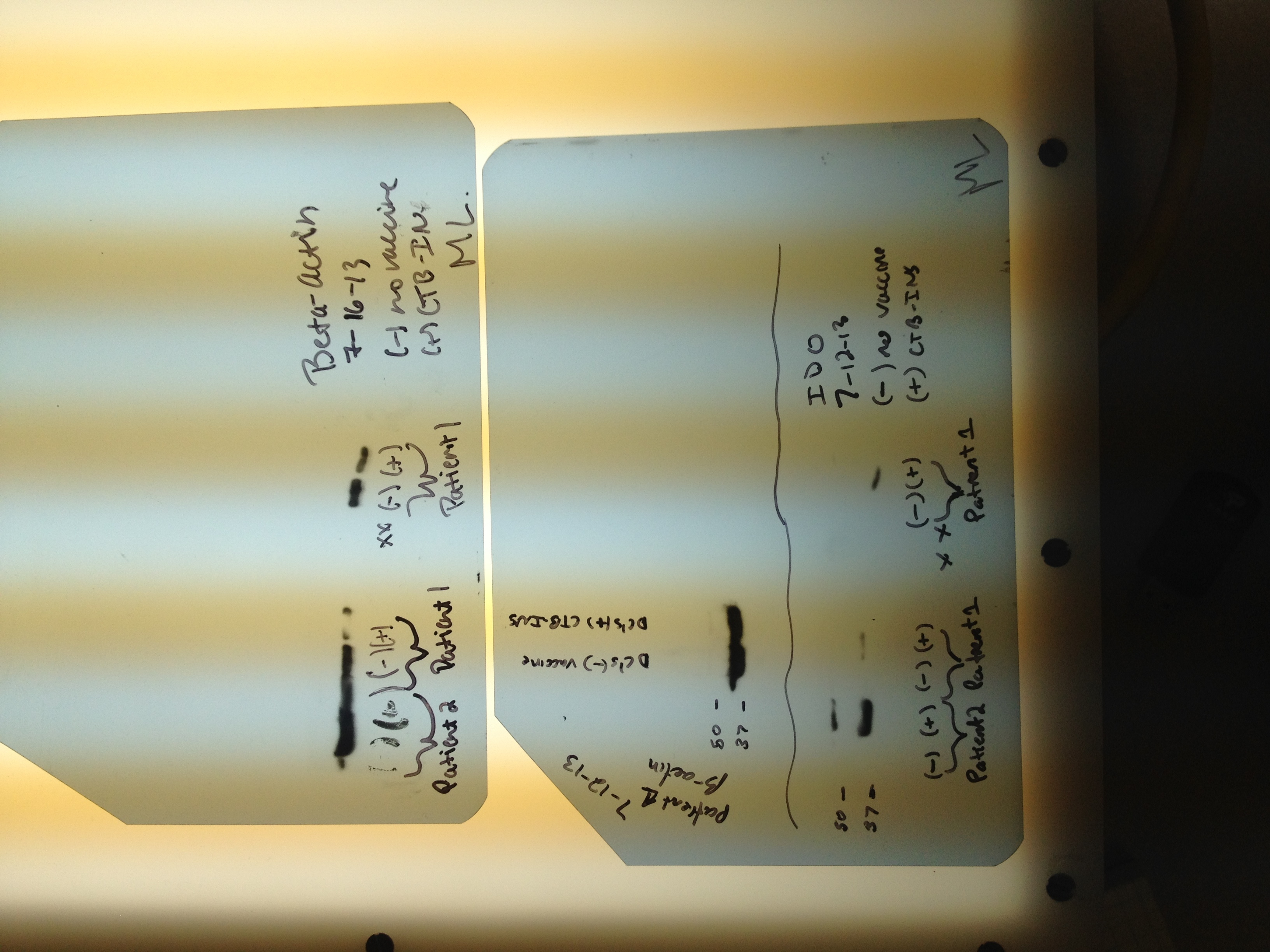

Supplement: S2 file — (JPG) [file pone.0118562.s002.JPG]

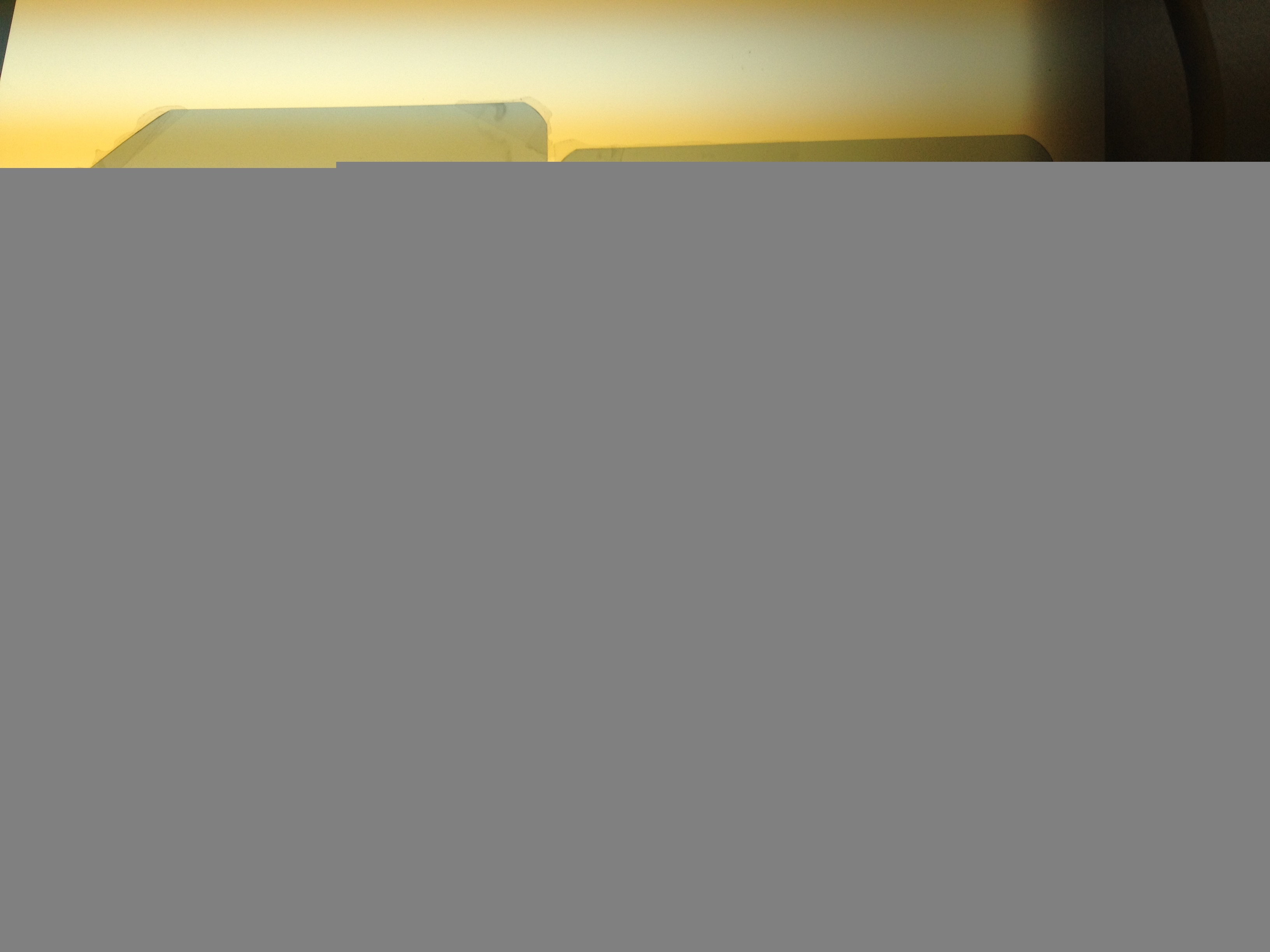

Supplement: S3 file — (JPG) [file pone.0118562.s003.JPG]

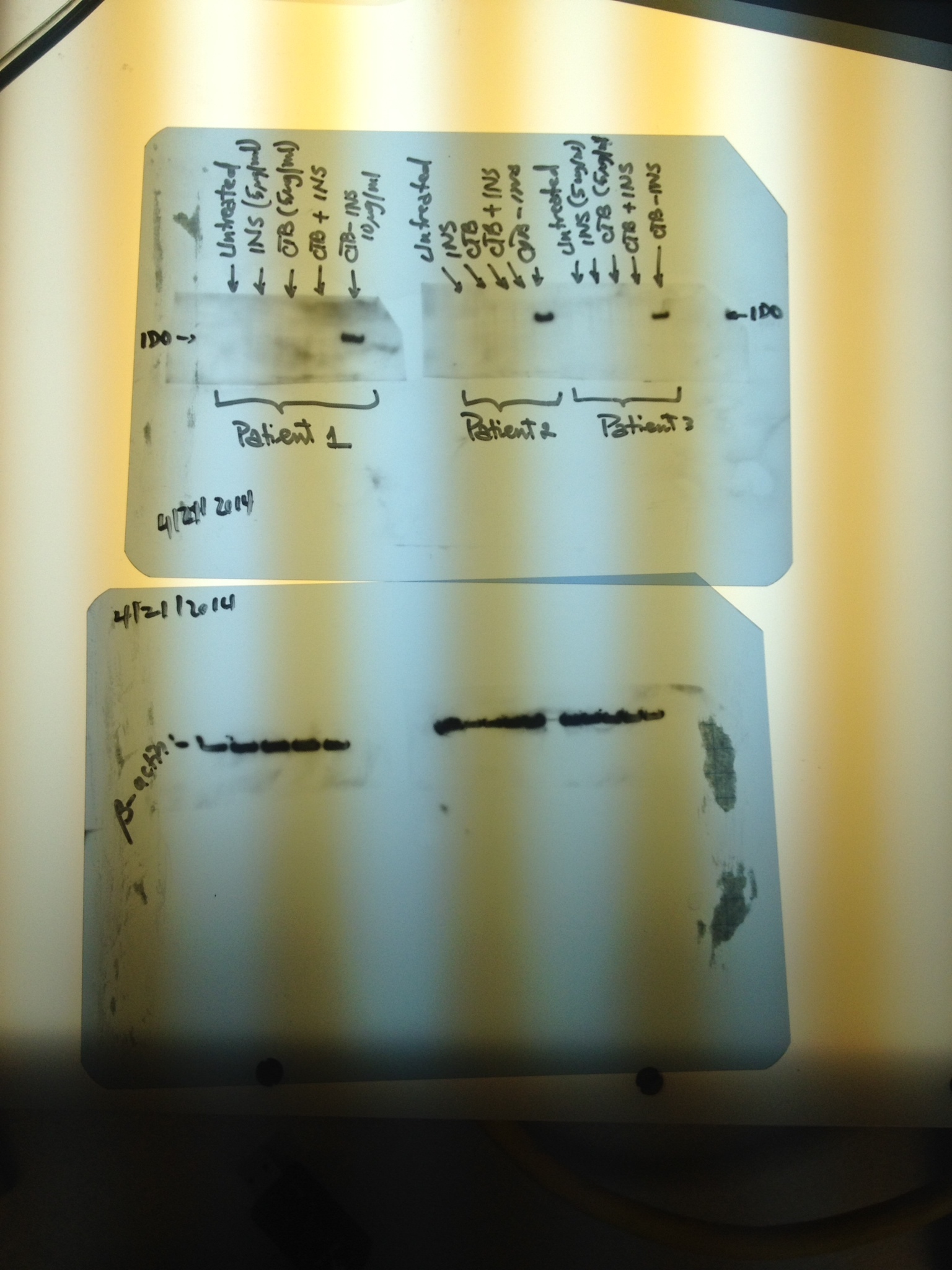

Supplement: S4 file — Three subjects represented. (JPG) [file pone.0118562.s004.JPG]

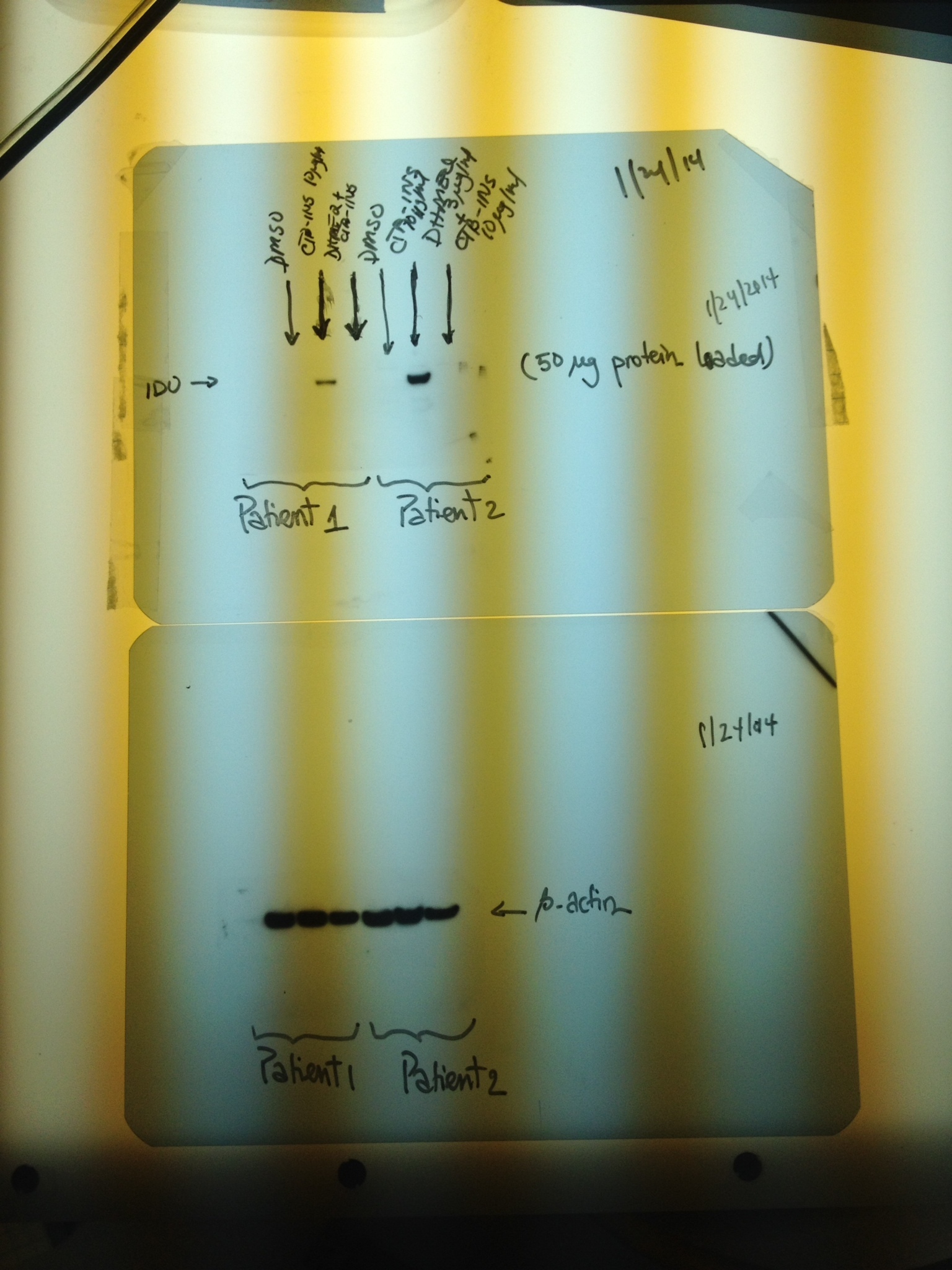

Supplement: S5 file — Two subjects shown. (JPG) [file pone.0118562.s005.JPG]

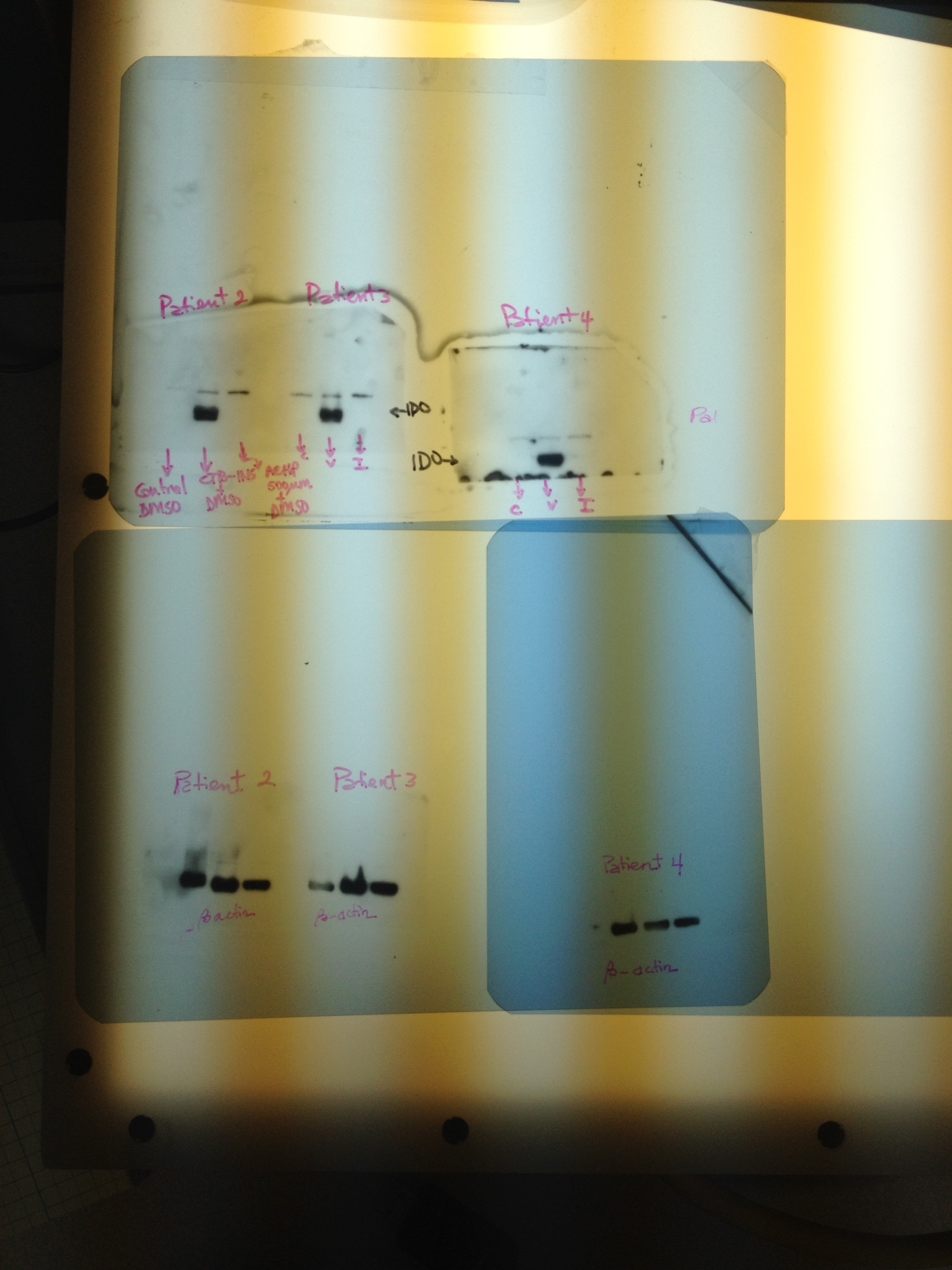

Supplement: S6 file — Three subjects shown. (JPG) [file pone.0118562.s006.JPG]
